# Supplementary material for: Therapeutic and Diagnostic Potential of Folic Acid Receptors and Glycosylphosphatidylinositol (GPI) Transamidase in Prostate Cancer
Source: Cancers (Basel). 2024 May 25;16(11):2008. doi: 10.3390/cancers16112008 (PMC11170984; doi:10.3390/cancers16112008)

Western Blot and Quantification

All quantifications were performed using ImageLab software. Background subtraction was applied to irregular backgrounds. Different positions of the quantification markers were always reviewed and provided comparable results. By using the stain-free protein gels, a loading control could be implemented on the total amount of protein effectively transferred to the PVDF membrane.

FR (Starbright 520) cell culture

Blot 1

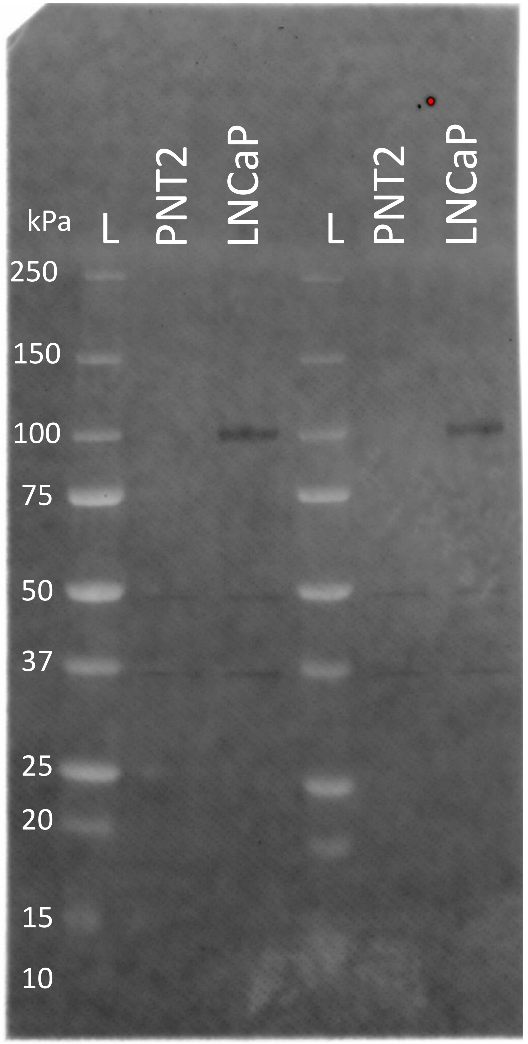

Quantification 1

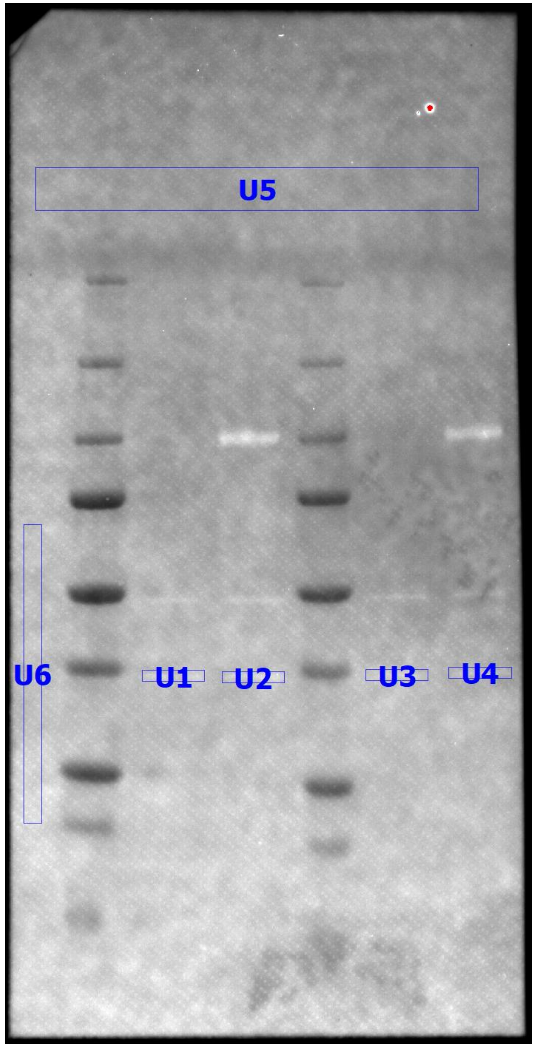

Blot 2

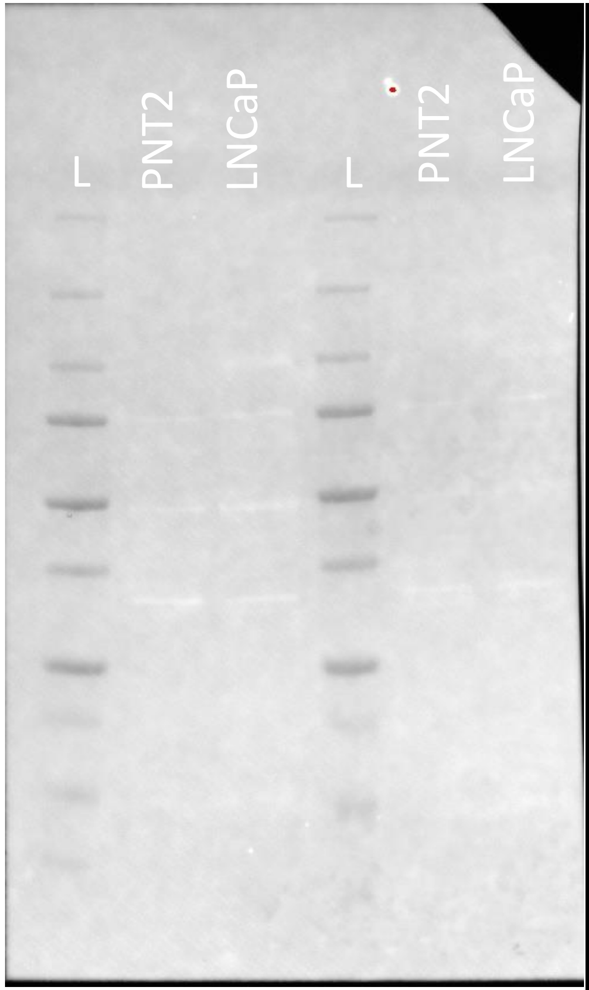

Quantification 2

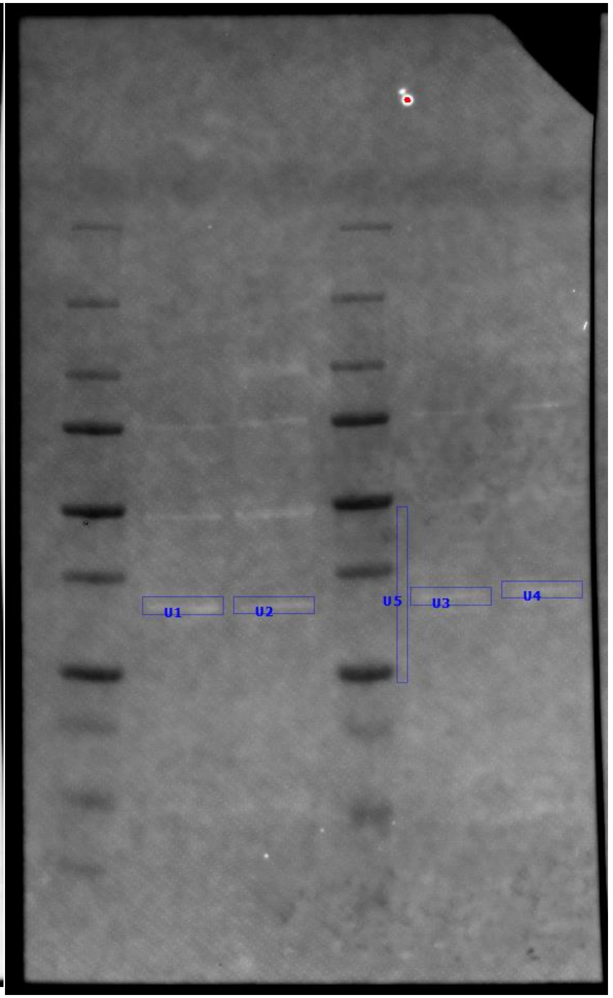

Quantification with bands 37 kDa and 50 kDa resulted in equal results!

GPI (Starbright 700) cell culture

Blot 1

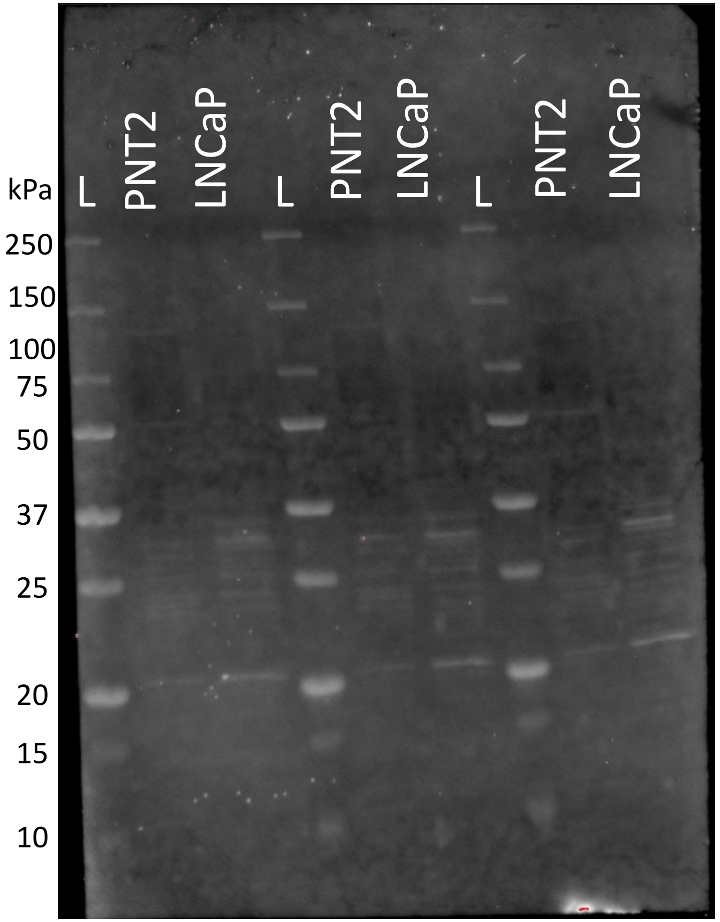

Quantification 1

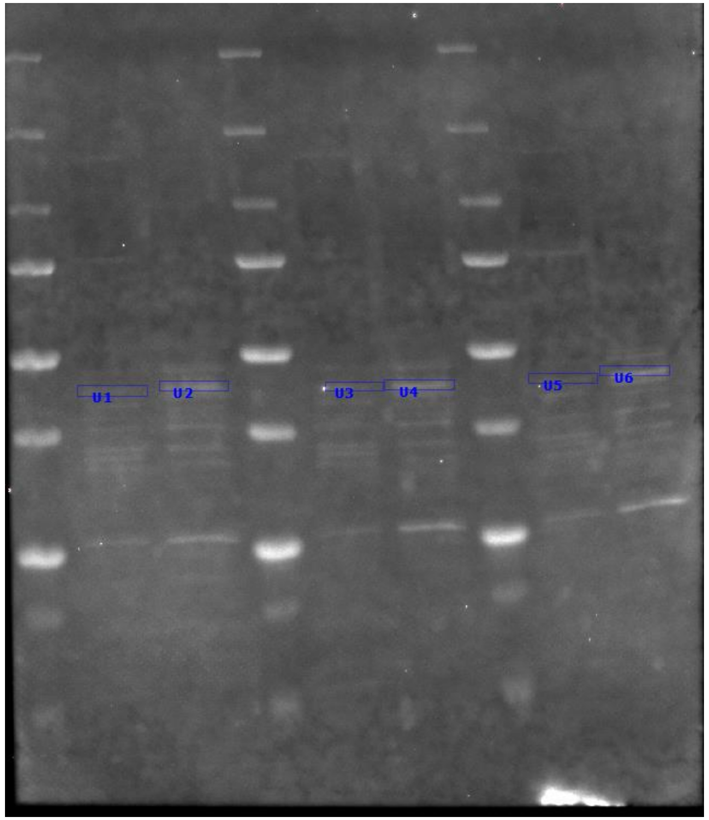

Blot 2

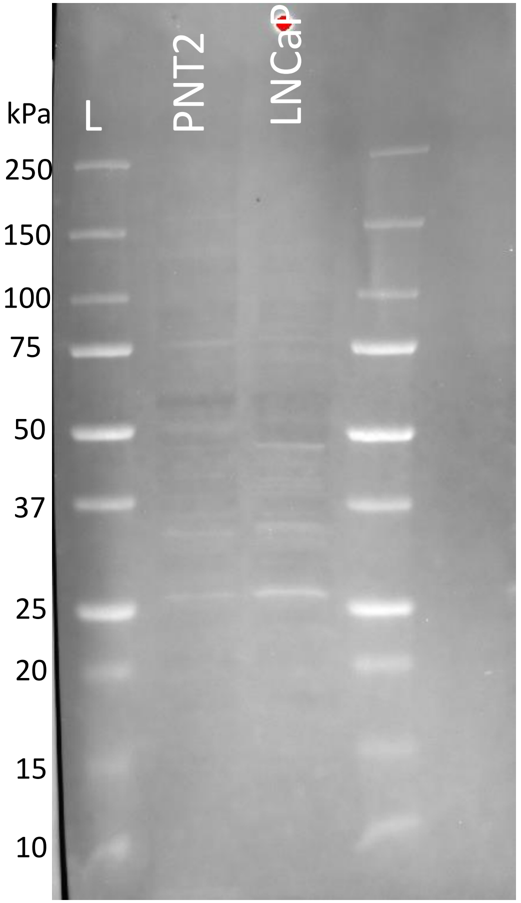

Quantification 2

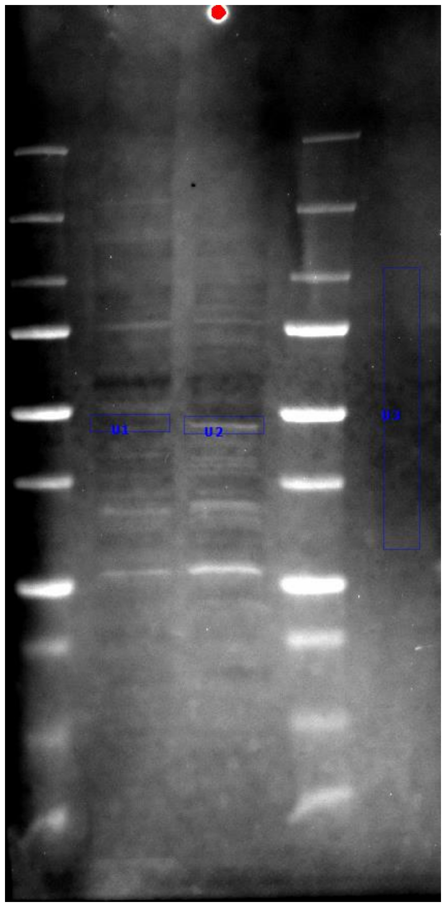

Stain free Protein load-control Quantification

Blot 1 / Quantification 1

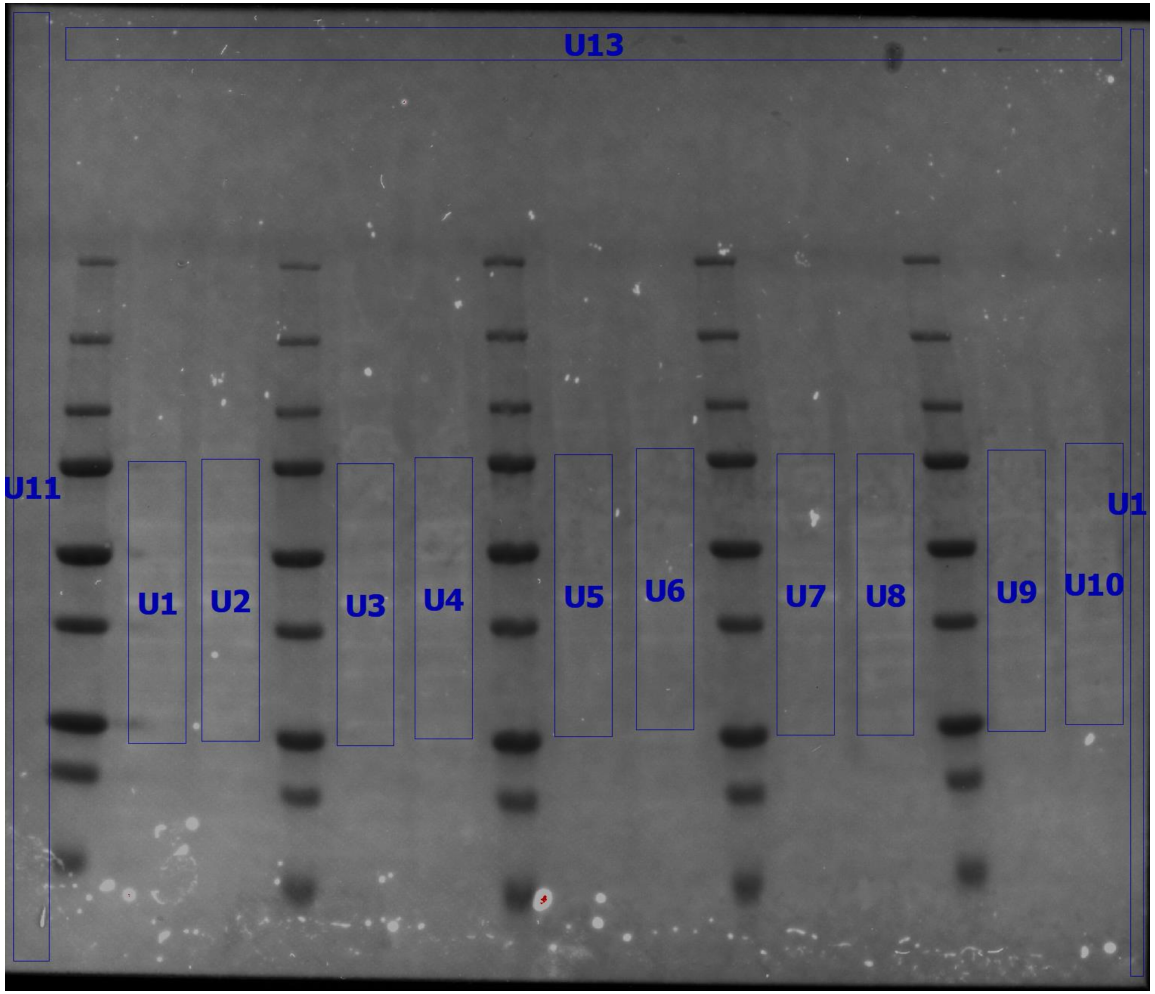

Blot 2 / Quantification 2

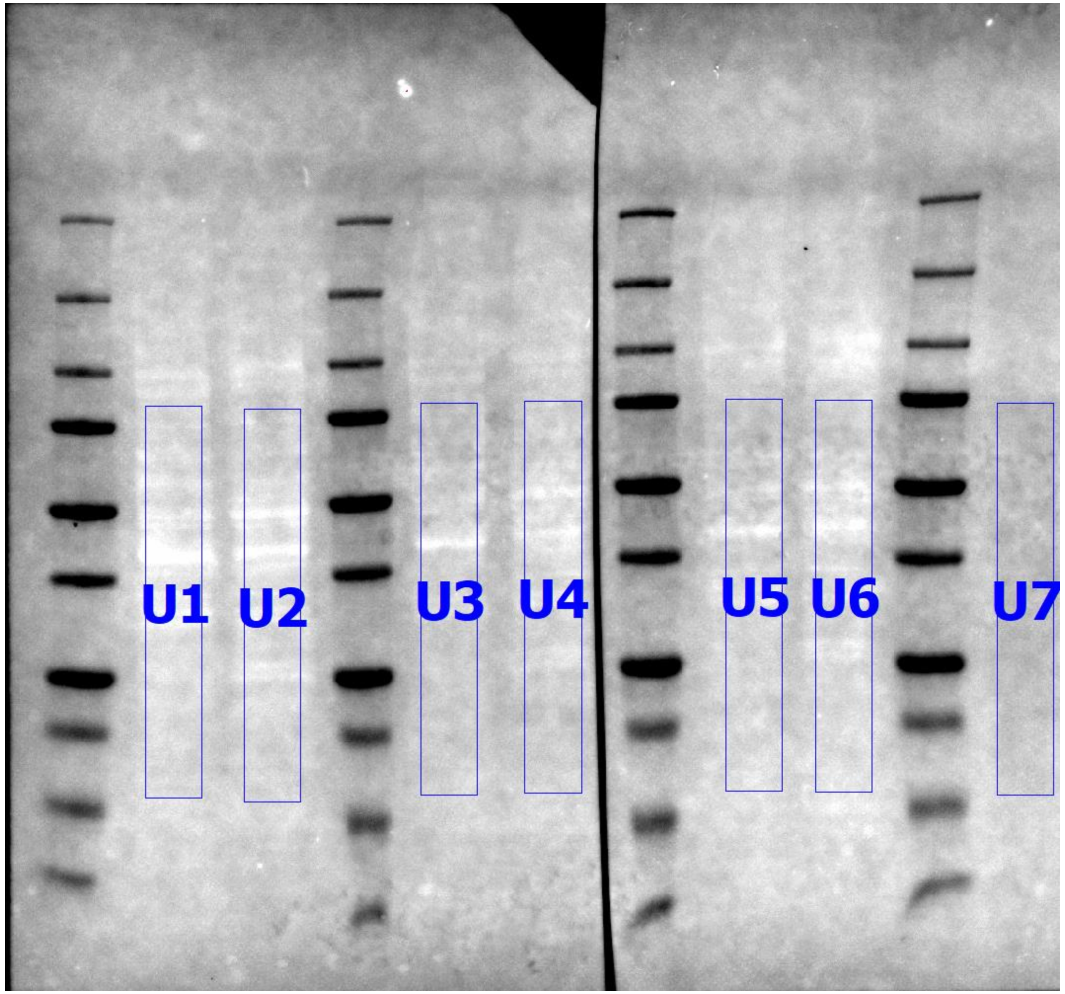

FR (Starbright 520) tissue samples

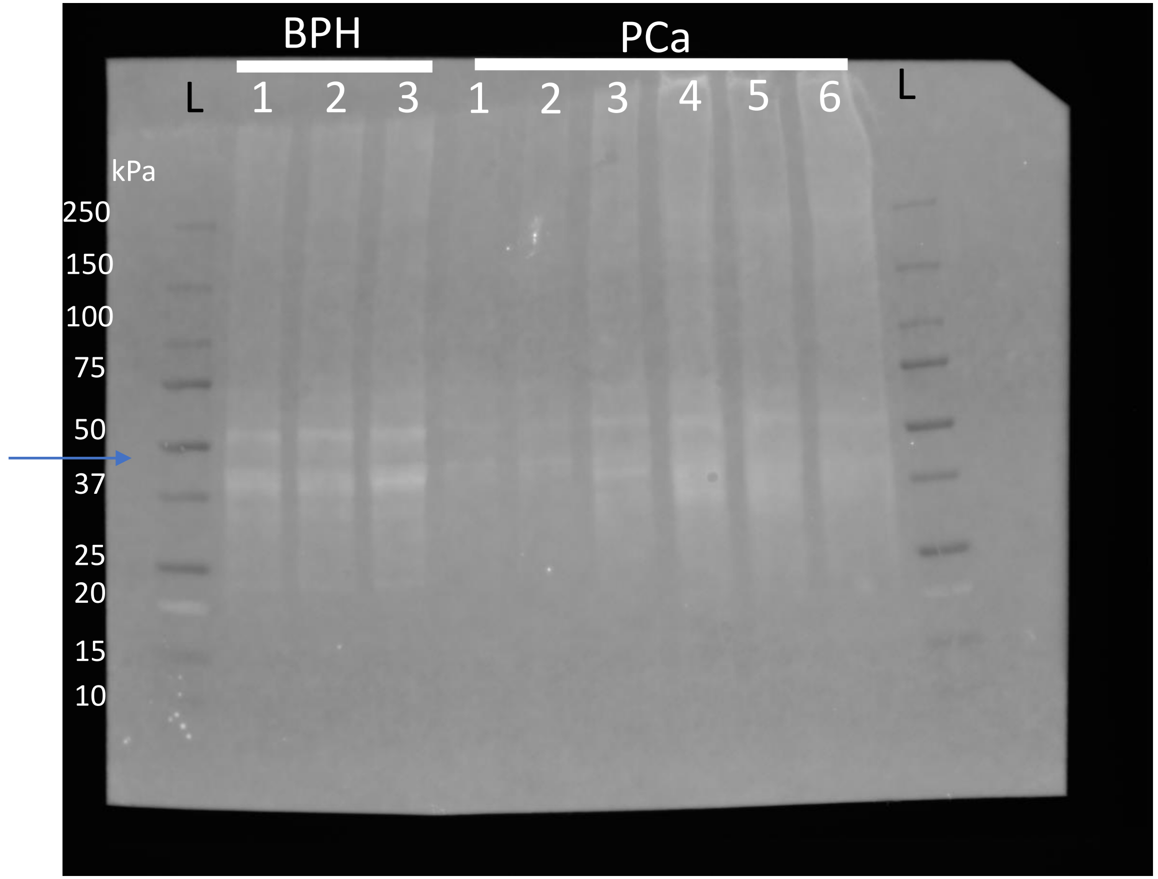

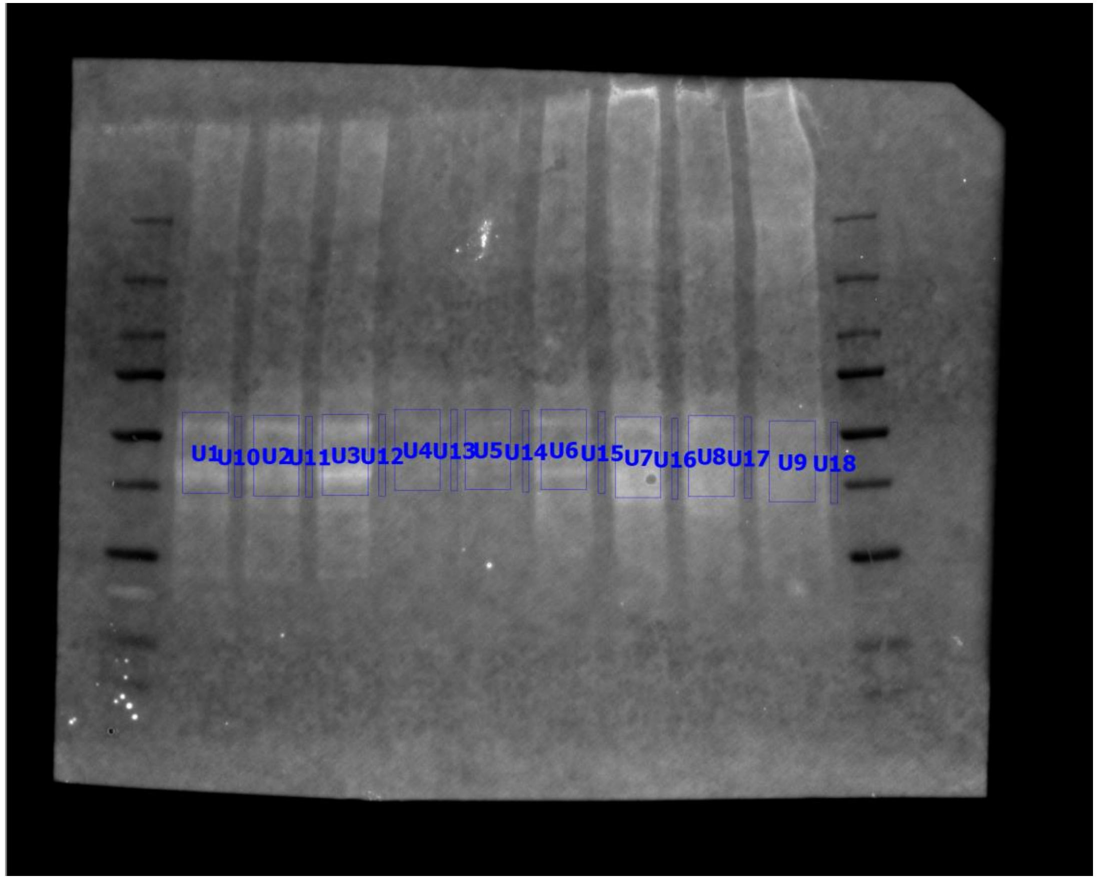

U1-U9 = Sample quantification
U10-U18 = Background quantification

Stain free Protein load-control Quantification

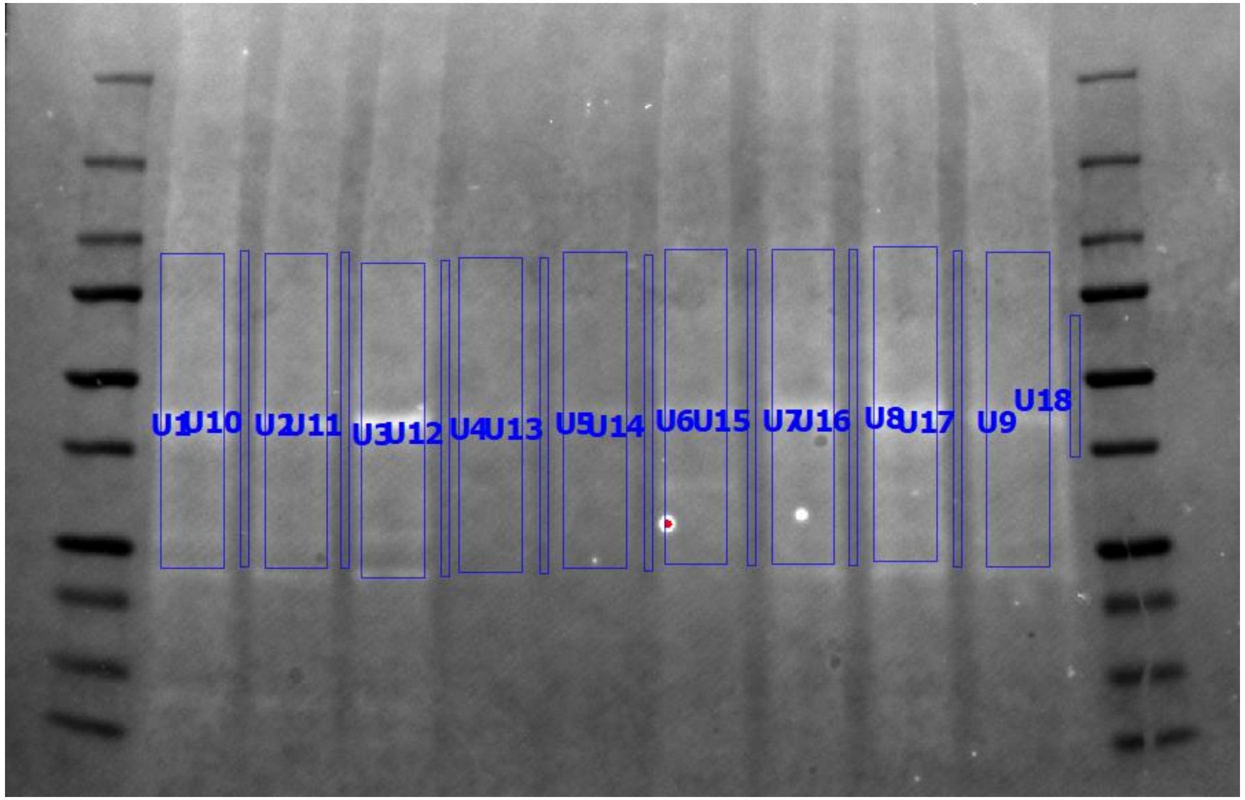

GPI (Starbright 700) Tissue samples

Degradet, specific band – included in quantification

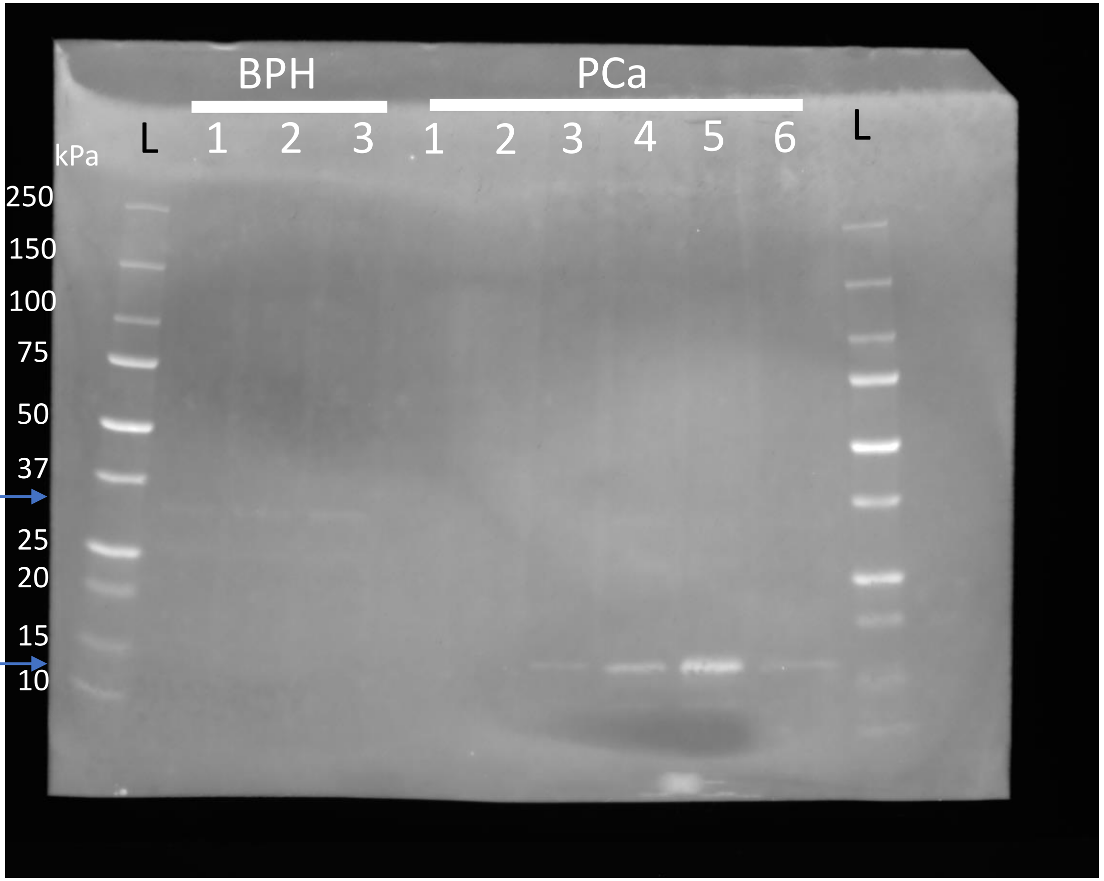

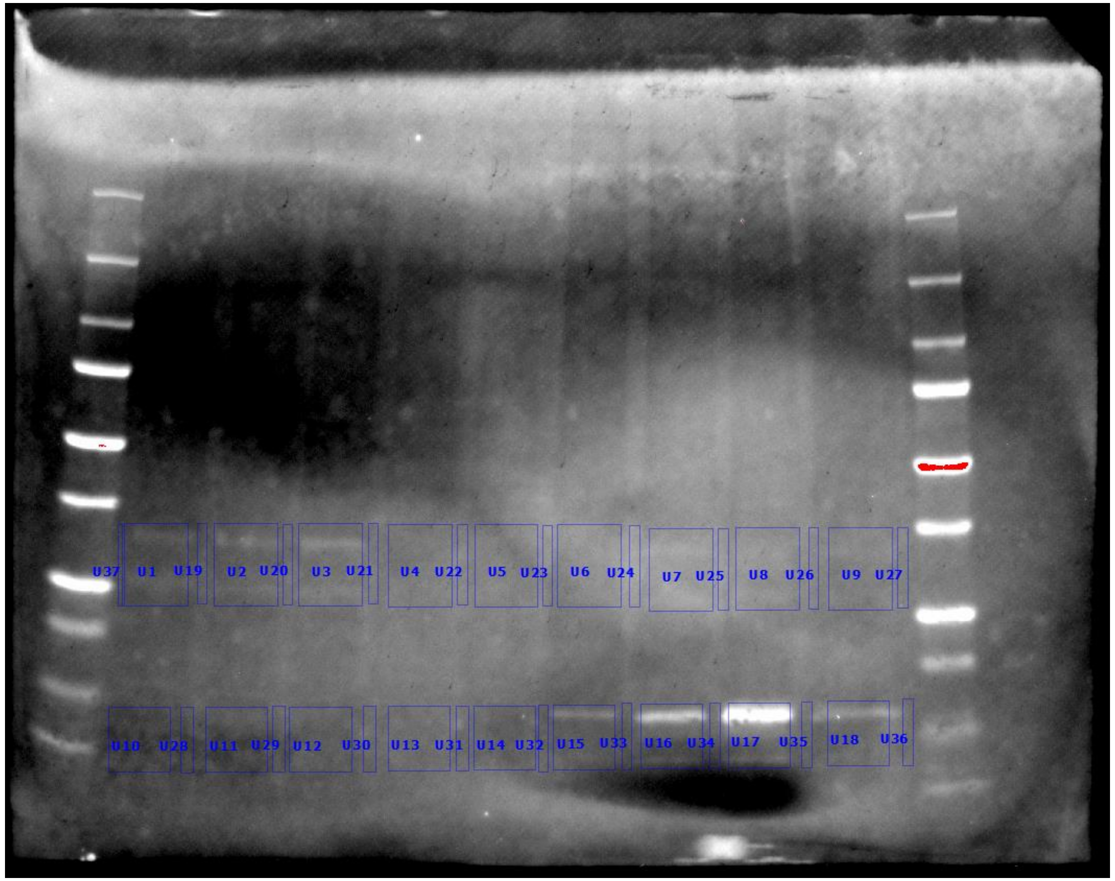

U1-U18 = Sample quantification
U19-U36 = Background quantification

Stain free Protein load-control Quantification

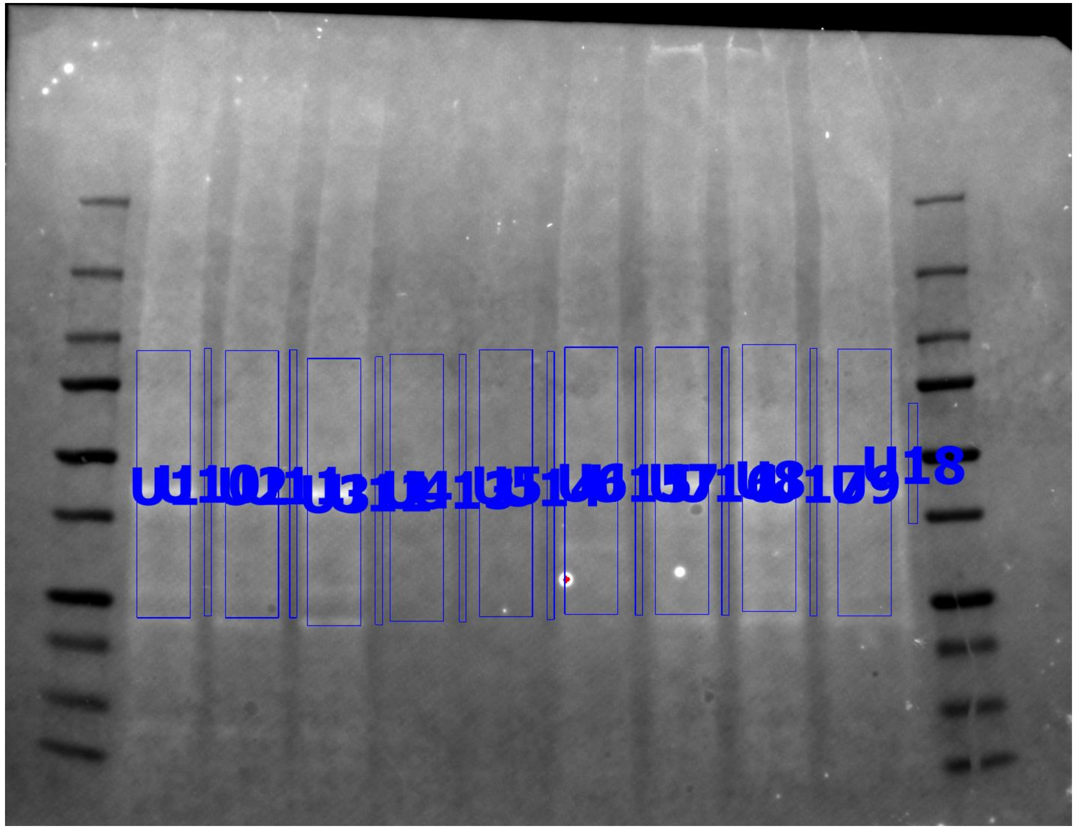

Supplement: Supplementary file 1 [file cancers-16-02008-s001.zip › Supplementary/Western Blots and Quantification.pdf]
