# Supplementary figures and images for: Therapeutic and Diagnostic Potential of Folic Acid Receptors and Glycosylphosphatidylinositol (GPI) Transamidase in Prostate Cancer
Source: Cancers (Basel). 2024 May 25;16(11):2008. doi: 10.3390/cancers16112008 (PMC11170984; doi:10.3390/cancers16112008)

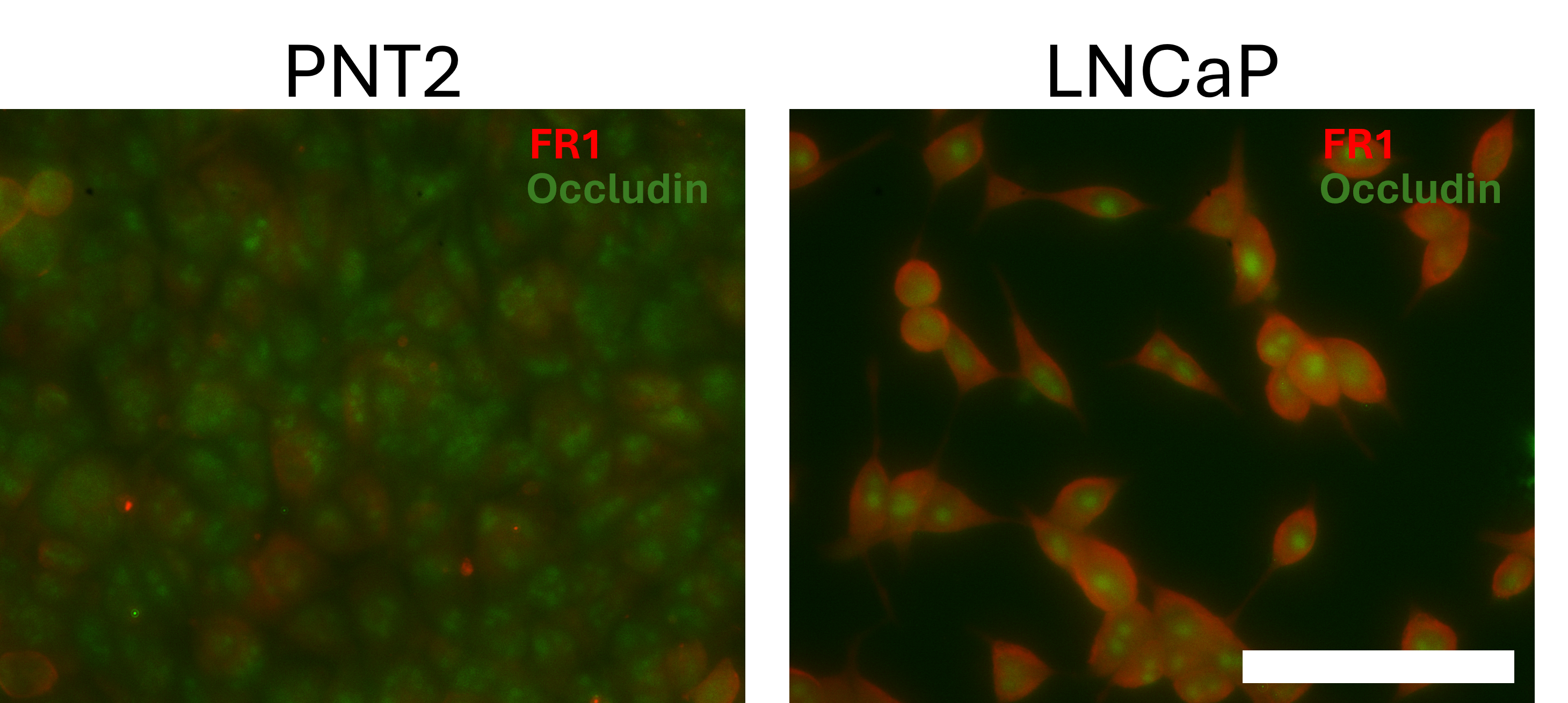

Supplement: Supplementary file 1 [file cancers-16-02008-s001.zip › Supplementary/Fig S1.png]

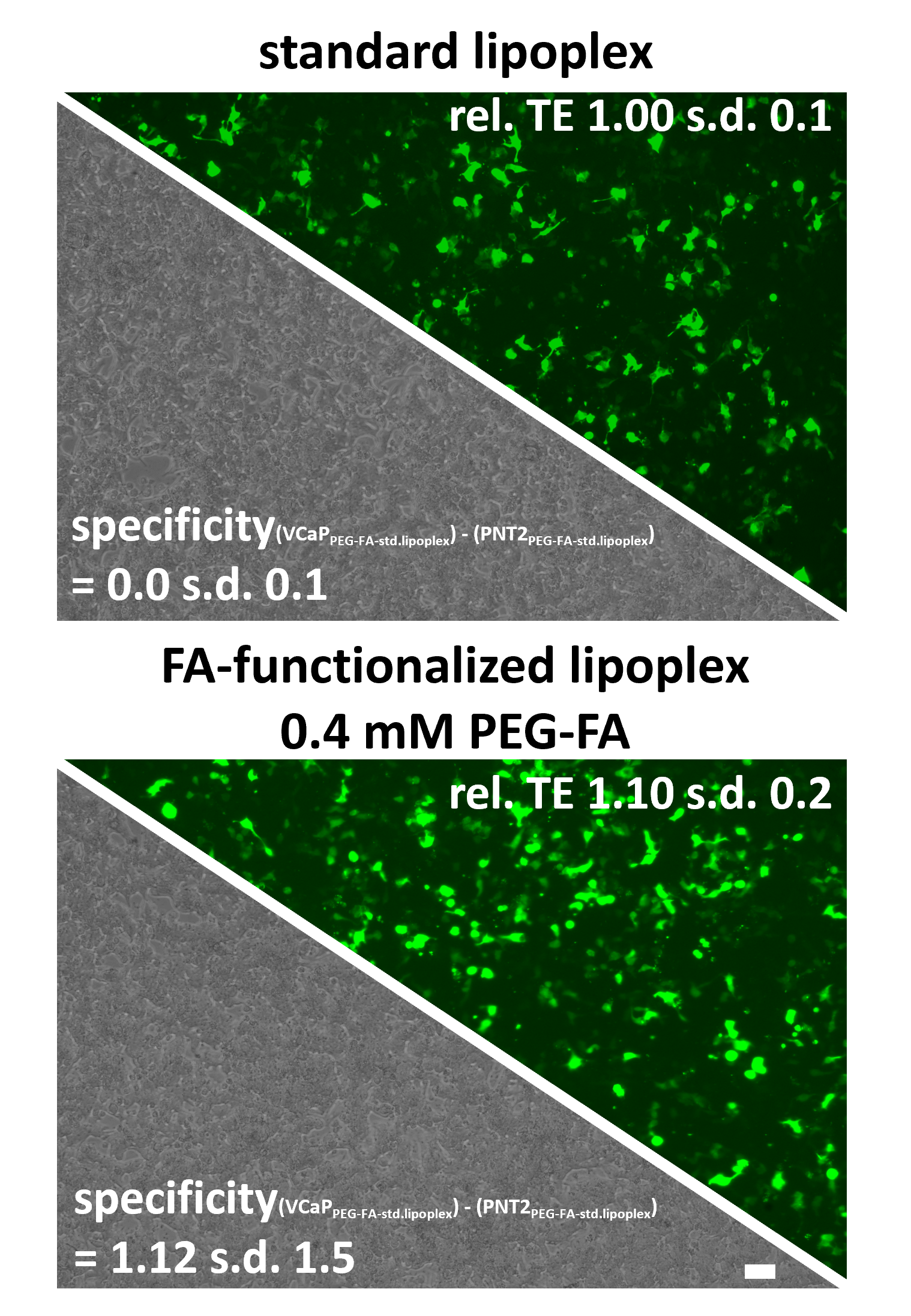

Supplement: Supplementary file 1 [file cancers-16-02008-s001.zip › Supplementary/Fig S2.png]

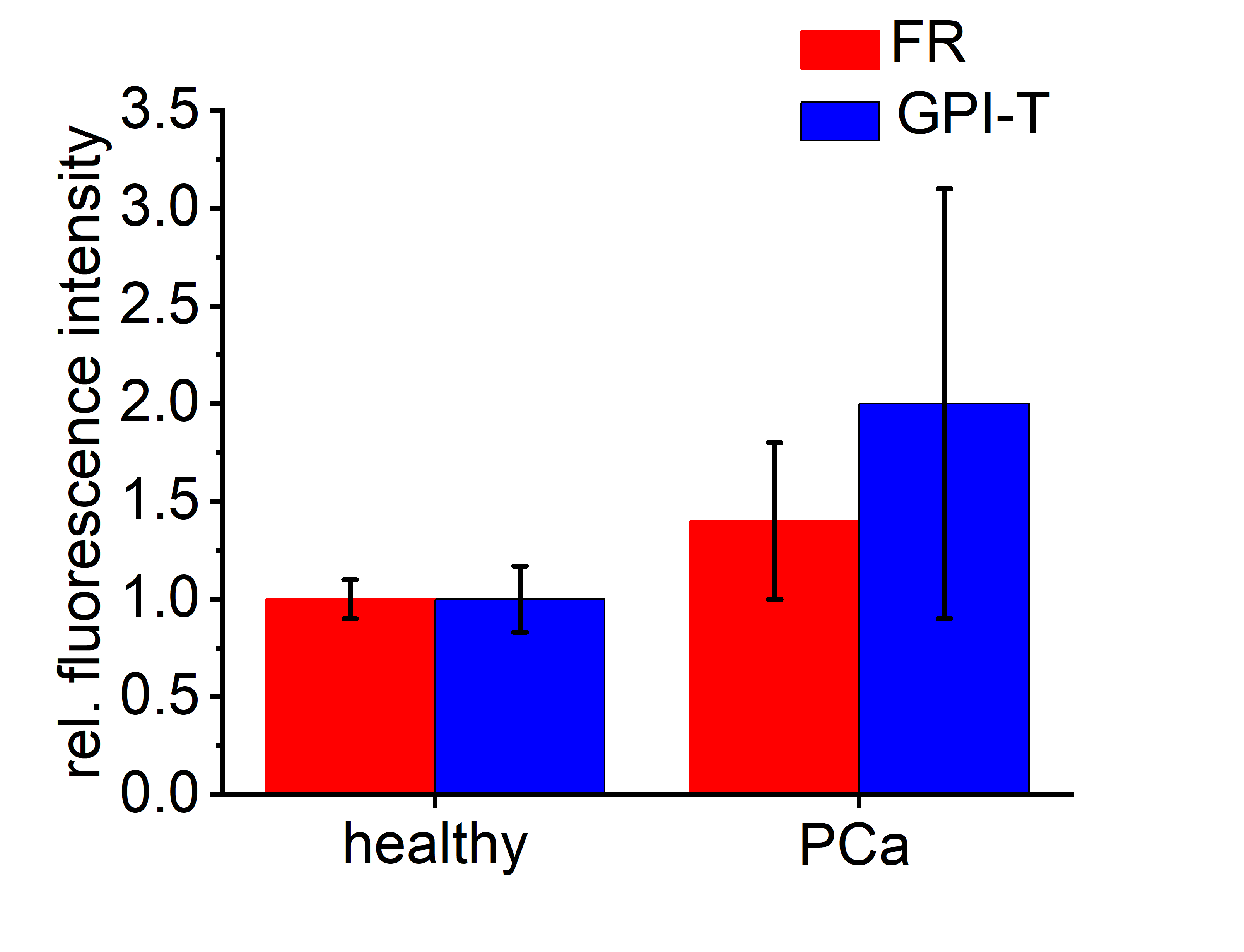

Supplement: Supplementary file 1 [file cancers-16-02008-s001.zip › Supplementary/Fig S3.png]
